# Whole Transcriptome Profiling of Successful Immune Response to *Vibrio* Infections in the Oyster *Crassostrea gigas* by Digital Gene Expression Analysis

**DOI:** 10.1371/journal.pone.0023142

**Published:** 2011-08-04

**Authors:** Julien de Lorgeril, Reda Zenagui, Rafael D. Rosa, David Piquemal, Evelyne Bachère

**Affiliations:** 1 Institut Français de Recherche pour l'Exploitation de la Mer, Centre National de la Recherche Scientifique, Montpellier, France; 2 Université Montpellier 2, and Institut de Recherche pour le Développement, UMR 5119 “Écologie des Systèmes Marins Côtiers”, Montpellier, France; 3 Skuld-Tech, Cap Delta, ZAC Euromedecine II, Grabels, France; French National Centre for Scientific Research - Université de Toulouse, France

## Abstract

The cultivated Pacific oyster *Crassostrea gigas* has suffered for decades large scale summer mortality phenomenon resulting from the interaction between the environment parameters, the oyster physiological and/or genetic status and the presence of pathogenic microorganisms including *Vibrio* species. To obtain a general picture of the molecular mechanisms implicated in *C. gigas* immune responsiveness to circumvent *Vibrio* infections, we have developed the first deep sequencing study of the transcriptome of hemocytes, the immunocompetent cells. Using Digital Gene Expression (DGE), we generated a transcript catalog of up-regulated genes from oysters surviving infection with virulent *Vibrio* strains (*Vibrio splendidus* LGP32 and *V. aestuarianus* LPi 02/41) compared to an avirulent one, *V. tasmaniensis* LMG 20012^T^. For that an original experimental infection protocol was developed in which only animals that were able to survive infections were considered for the DGE approach. We report the identification of cellular and immune functions that characterize the oyster capability to survive pathogenic *Vibrio* infections. Functional annotations highlight genes related to signal transduction of immune response, cell adhesion and communication as well as cellular processes and defence mechanisms of phagocytosis, actin cytosqueleton reorganization, cell trafficking and autophagy, but also antioxidant and anti-apoptotic reactions. In addition, quantitative PCR analysis reveals the first identification of pathogen-specific signatures in oyster gene regulation, which opens the way for in depth molecular studies of oyster-pathogen interaction and pathogenesis. This work is a prerequisite for the identification of those physiological traits controlling oyster capacity to survive a *Vibrio* infection and, subsequently, for a better understanding of the phenomenon of summer mortality.

## Introduction

Aquatic organisms and particularly marine invertebrates, such as the oyster *Crassostrea gigas*, harbour an abundant and diverse microflora on their surface (epibiosis) or inside their tissues (endobiosis) where *Vibrio splendidus* is found as a dominant culturable vibrio. With Evolution, the oysters have developed effective systems for maintaining their homeostasis and for controlling potentially harmful and pathogenic bacteria. However, for decades, the cultivated Pacific oyster *C. gigas* is suffering large scale summer mortalities that are reported in all areas of the world where this species is cultivated [1]. Mortalities result from the interaction between the environment, the oyster physiological and/or genetic status and the presence of pathogenic microorganisms [1]. Beside the recent identification of a virulent microvariant of an Herpes virus, the OsHV-1 [2], *Vibrio* strains of *V. splendidus* and *V. aestuarianus* groups have been repeatedly associated to summer mortality episodes [3] and the virulence of some strains has been demonstrated through *C. gigas* experimental infections [4], [5].

Considerable effort has been invested in advanced genomic technologies to understand and characterize the major traits that govern the tolerance of oysters to stressful culture conditions or to pathogenic bacteria [6], [7], [8], [9], [10], [11]. In particular, immune-related genes have been characterized from *C. gigas.* Briefly, a variety of antimicrobials have been fully characterized, namely a Bactericidal/Permeability-Increasing protein, *Cg*-BPI [12] and antimicrobial peptide families, *Cg*-Defensins (*Cg*-Defs) [13], *Cg*-Proline rich peptides (*Cg*-Prps) [14], and a great diversity has been demonstrated in terms of sequences and potential antimicrobial activities [15]. Tissue Inhibitor Metalloprotease (TIMP)-encoding gene has been shown to be induced upon microbial challenge, the expression of which would be controlled by a Rel/NF-κB pathway [16], [17]. The oyster major plasma protein, *Cg*-EcSOD (extracellular Superoxide Dismutase) which appears to display anti-oxidant and LPS-binding properties [18] has recently been shown to be used as an opsonin for *V. splendidus* LGP32 phagocytosis through its RGD sequence [19]. Otherwise, oyster infection with *V. aestuarianus* results in a decrease in *Cg*-EcSOD transcripts in circulating hemocytes [20]. However, whereas most of these immune genes were shown to be modulated during infections, the molecular mechanisms by which the oyster can survive virulent *Vibrio* infections remained totally unknown.

Here, our objective was to develop a better understanding of the genetic-level responses of oysters to pathogenic vibrios and to identify genes that are involved in immune responsiveness to circumvent the infections. In this attempt, we have performed a comprehensive analysis of the transcriptome of oyster immunity (hemocytes), using Digital Gene Expression (DGE) [21], an improved version of the Serial Analysis of Gene Expression (SAGE) technique [22]. These methods generate genome-wide and high-throughput transcription profiles and provide qualitative and quantitative gene expression data that do not depend on prior identification of transcript information (sequence homologies or gene function). For the DGE approach, we have developed an original experimental infection protocol, considering individual monitoring of the oyster successful response in terms of survival to pathogenic *Vibrio* infections *versus* non pathogenic ones. Thus, two DGE libraries were constructed from hemocytes of oysters surviving infections by virulent *V. splendidus* LGP32 and *V. aestuarianus* LPi 02/41 on the one hand, and by *V. tasmaniensis* LMG 20012^T^, an avirulent strain related to the *V. splendidus* group, on the other hand. The study aimed to compare the expression data of the two libraries and, beyond gene identification and functional annotation, to explore the putative functions related to the capability of oysters to circumvent and to survive vibrioses. This is the first report on genome-wide transcriptional analysis of oyster survival-responsiveness to virulent vibrios.

## Results

### Oyster survival to virulent *Vibrio* species

**Figure 1:**
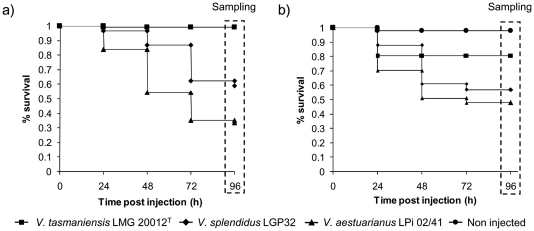
Kaplan-Meier survival curves of *C. gigas* oysters during infections with virulent *Vibrio* strains, *V. aestuarianus* LPi 02/41 or *V. splendidus* LGP32, and with avirulent *V. tasmaniensis* LMG 20012^T^. Infections for (a) construction of the hemocyte DGE libraries and (b) for gene expression exploration by qPCR analysis, with non injected oysters as control. For the two experiments, hemolymph was individually sampled from oysters that survived the infection at 96 h post-infection corresponding to the end of mortalities.

To strengthen the accuracy of our transcriptomic approach, two independent experimental infections have been performed for (i) the construction of hemocyte DGE libraries and for (ii) the exploration of the DGE data by qPCR, using respectively oysters from the Atlantic coast and Mediterranean Lagoon that differ in abiotic and biotic environmental conditions. After infections, the mortalities were individually monitored and Kaplan–Meier survival curves were generated for the different groups of oysters (**Figure 1**).

For the Atlantic oysters, the survival rates were 58.9% for the animals infected by virulent *V. splendidus* LGP32 (8.10^7^ CFU/oyster), 33.3% for the oysters infected with the highly virulent *V. aestuarianus* LPi 02/41 (2.10^7^ CFU/oyster) and 96.9% for those injected with the avirulent *V. tasmaniensis* LMG 20012^T^. The peaks of mortalities were reached at 72 h and 48 h post-injection respectively for the *V. splendidus* and *V. aestuarianus* strains (**Figure 1a**). Kaplan–Meier survival curves revealed statistical differences between the oysters injected either with *V. splendidus* LGP32, *V. aestuarianus* LPi 02/41 or the avirulent strain (*p*<0.0001, Log-Rank and Wilcoxon tests). For the Mediterranean Lagoon oysters, the two groups of individuals infected with *V. splendidus* LGP32 (4.10^8^ CFU/animal) or *V. aestuarianus* LPi 02/41 (8.10^7^ CFU/animal) displayed respectively 57% and 48% survival, with peaks of mortalities reached at 48 h post-infection for both bacteria strains. For the third group injected with *V. tasmaniensis* LMG 20012^T^ (2.10^8^ CFU/animal), 80% survival was observed at 24 h post-injection whereas 98% survival were recorded for the fourth non-injected oyster group used as control **(** **Figure 1b** **)**. Generated Kaplan–Meier survival curves revealed significant differences (*p*<0.0001, Log-Rank and Wilcoxon tests, DDL = 2) between the four experimental infections. The survival curves differed statistically between the two independent infections (*p*<0.0001, Log-Rank and Wilcoxon tests, DDL = 5), but no differences were observed between Atlantic oysters injected with *V. tasmaniensis* and non injected Mediterranean oysters (*p*>0.05, Log-Rank and Wilcoxon tests, DDL = 5). We showed that higher dose of *V. splendidus* LGP32 (4.10^8^ CFU/oyster) was requested for Mediterranean oysters than for the Atlantic ones (8.10^7^ CFU/oyster) to reach similar mortality rates. This would confirm variabilities previously observed in oyster susceptibility to infections according to their geographic origins (Duperthuy, pers. comm.).

To consider only animals that have been able to survive infections, hemolymph for hemocyte RNA extraction was individually collected at 96 h post-infection when mortalities were no more recorded.

### General characteristics of hemocyte DGE libraries

To have access to the hemocyte transcriptome and to establish a complete quantitative and qualitative gene expression database for the oyster immune functions and survival to virulent vibrios, two DGE libraries were generated from pooled hemocyte RNAs of the Atlantic oysters (i) surviving virulent *V. splendidus* LGP32 and *V. aestuarianus* LPi 02/41 infections (SVir library) and (ii) those challenged with the avirulent strain, *V. tasmaniensis* LMG 20012^T^ (SaVir library). Sequencing of these two libraries resulted in a total of 6,983,680 DGE tags. Characteristics of the libraries are summarized in **Table 1**. According to P-value (0.001) and occurrence of tags (incidence of each tag in libraries) in the DGE libraries, 56,871 unique tags have been identified from both libraries (GEO accession numbers GSM667899 and GSM667900). Comparison of the tag occurrences between libraries revealed that 22,187 unique tags are differentially represented between libraries (fold change >2), composed of 9,815 tags more represented in SVir library and 12,372 tags more represented in SaVir library. By using Blast search on the *C. gigas* 29,745 unique ESTs (http://www.sigenae.org/aquafirst/), these DGE tags were found to be associated to 4,374 ESTs corresponding to 3,931 unique ESTs (1,610 for SVir and 2,321 for SaVir), which have been further considered for functional annotation of genes related to survival response to virulent *versus* non virulent vibrios. The relatively low percentage of homology found (19.7%) is mainly due to the fact that the ESTs assembled in the GigasDatabase were obtained from different developmental stages and oyster tissues that include only 13,898 ESTs from hemocytes [10].

**Table 1:** General characteristics of DGE libraries generated from oysters surviving virulent (SVir) and avirulent (SaVir) *Vibrio* infections.

DGE library	SVir	SaVir
Sequenced tags	3,349,884	3,633,796
Unique tags	52,224	52,528
Specific tags	3,647	4,343
Tags differentially represented (≥2 fold change)	9,812	12,372
Number of tags which match with EST	1,782	2,592
Unique EST	1,610	2,321
EST with B2GO annotation	393	766
EST with KEGG annotation	271	564

### Exploration of differential gene expressions according to *Vibrio* strains

**Figure 2:**
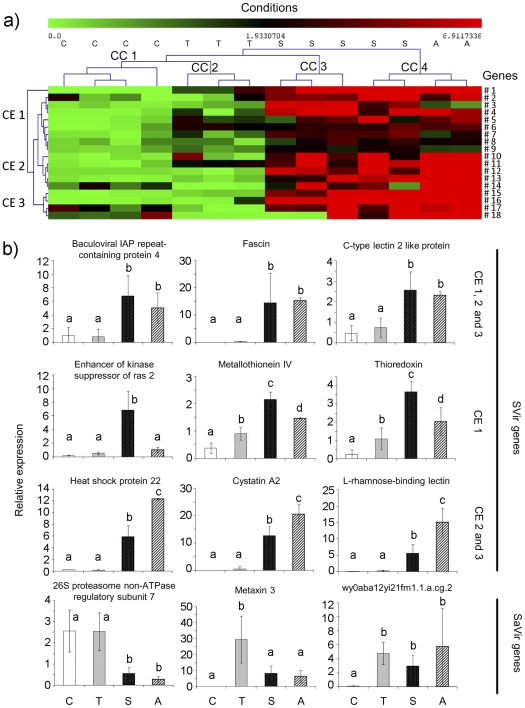
Gene expression analysis by qPCR. (a) *Hierarchical clustering* analysis and differential expression of 18 genes from SVir library. Hemocyte gene expression profiles were analysed in biological replicates from oysters: non injected as control (C), surviving infections with avirulent *V. tasmaniensis* LMG 20012^T^ (T), with virulent *V. splendidus* LGP32 (S) and virulent *V. aestuarianus* LPi 02/41 (A). Each biological replicata was constituted by a pool of hemocyte RNA from ten oysters, and between 2 and 5 replicates were analysed for each experimental condition. Each cell in the matrix corresponds to the expression level of one gene in a sample. The intensity of the color from green to red indicates the magnitude of differential expression (see color scale at the bottom of the image). Relative expressions were calculated according the 2^−(ΔΔCt)^ method normalized with elongation factor-1α (EF-1α). Each value was calculated in reference to the mean of ΔCt of all conditions (relative expression = 1). The dendrograms at the top of the figures indicate relationship among experimental conditions which define clusters of conditions (CC). The dendrograms at the left of the figures indicate relationship among the profiles of the selected genes which define clusters of expression (CE), after clustering analysis using Multiple Array Viewer software. Gene #1: Inhibitor of apoptosis; #2: Baculoviral IAP repeat-containing protein 4; #3: Enhancer of kinase suppressor of Ras2; #4: Rac GTPase-activating protein1; #5: Proteasome 216S subunit, non-ATPase 11a; #6: Glyceraldhyde 3-phosphate dehydrogenase; #7: Thioredoxin; #8: C-type lectin 2 like protein; #9: Metallothionein IV; #10: F-box only protein37; #11: Cystatin B-like protein; #12: Heat shock protein 22 isoform 1; #13: L-rhamnose-binding lectin; #14: Microsomal glutathione S-tranferase; #15: Cystatin A; #16: Interferon-induced protein 44; #17: Cullin-associated and neddylation-dissociated 1. Hierarchical clustering was contructed with Multiple Array Viewer software using average linkage clustering with Pearson correlation as the default distance metric. (b) *Examples of gene expression profiles defining pathogen- or challenge-specific signatures*. SVir genes; line 1: similar response to both virulent strains; line 2: response induced by *V. splendidus* LGP32; line 3: response induced by *V. aestuarianus* LPi 02/41; line 4: SaVir genes. Different letters indicate significant variation between conditions (*p*<0.05) determined using the non-parametric multiple comparison test ANOVA of Kruskal-Wallis.

The accuracy of our DGE approach have been validated by qPCR analysis. To reinforce this exploration, we used an independent experimental infection with oysters from Mediterranean Lagoon instead of Atlantic coast. For that, 18 genes more represented in SVir library and 17 genes more represented in SaVir library (2-fold change) were randomly chosen. Transcript levels were measured from hemocytes of oysters that survived infections with the virulent strains and the avirulent control. In addition, non-injected oysters were used as a control group (**Figure 1b**). Hierarchical clustering of the 18 SVir gene expression profiles distinguished, through clusters of conditions (CC), surviving oysters to virulent *Vibrio* strain infections (CC 3 and 4) to injected oysters with avirulent *Vibrio* strain (CC 2) and non injected oysters (CC 1) with a range of differential expression of 6.9-fold (**Figure 2a**). We can notice than up-regulated genes were found in all clusters of expression (CE) independently of the virulent *Vibrio* strains injected. Comparatively expression profiles of the 17 SaVir genes did not discriminate oyster response according to the *Vibrio* strains or control in the clustering (**Figure S1**). Furthermore, for these genes, a 2.6-fold change of differential expression was observed, weaker than obtained for SVir genes.

### Pathogen-specific signatures are evidenced among DGE genes

It is noteworthy that hierarchical clustering evidenced pathogen-specific and shared signatures upon bacterial challenges. Three major clusters of expressions (CE) were evidenced for SVir genes. Indeed, we observed genes up-regulated only in response to both virulent *Vibrio* compared to control or avirulent *Vibrio* injection (as examples: baculoviral IAP repeat containing protein 4, fascin or C-type lectin). Besides, genes were seen to be up-regulated in response to *V. splendidus* associated to cluster of expression 1 (as examples: Enhancer of kinase suppressor of ras 2, Metallothionein IV and Thioredoxin), while up-regulated genes in response to *V. aestuarianus* infection were found in clusters of expression 2 and 3 (as examples: Heat shock protein 22, Cystatin A2 and L-rhamnose-binding lectin) (**Figure 2b** **)**. Various expression profiles were found for the SaVir genes tested, such as a down-regulation upon virulent *Vibrio* infections (as an example, the putative 26S proteasome non-ATPase regulatory subunit 7), up-regulation by the avirulent strain compared to virulent strains (Metaxin 3) or up-regulated indistinctly by the avirulent and virulent *Vibrio* compared to unchallenged oysters (no Blast hit sequence wy0aba12yi21fm1.1.a.cg.2).

### Functional annotation

**Figure 3:**
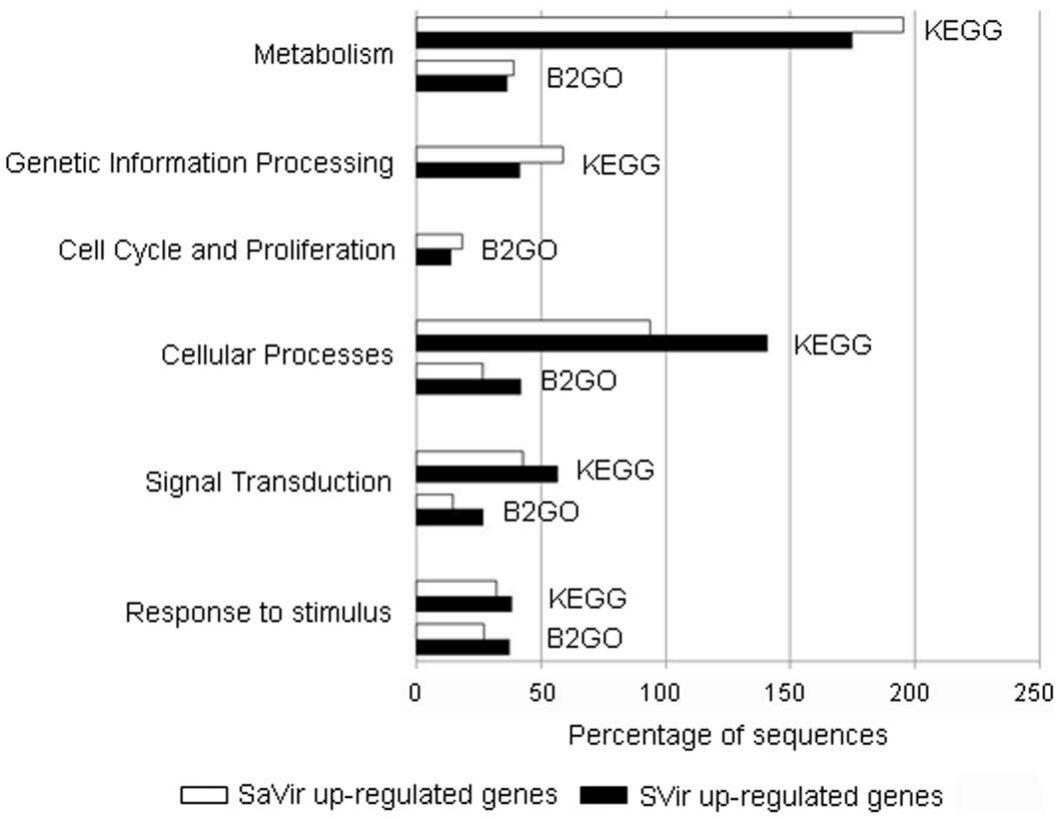
Functional annotation of unique ESTs differentially represented between SVir and SaVir libraries, respectively. Categorization was based on KO terms of the Kyoto Encyclopedia of Genes and Genomes (KEGG) using KEGG Automatic Annotation Server (KAAS), and on GO terms of Biological Process using Blast2GO software.

Altogether, the qPCR analyses which were performed on independent experimental infections and distinct biological material corroborated the DGE quantitative data. This supported further bioinformatic and Blast2GO and KEGG annotations [23] aiming to gain a general picture of the functional profiles of oyster survival- and infection-responsive genes identified by DGE approach. The 3,931 unique ESTs differentially represented between libraries (2-fold change) were annotated into 7 and 5 functional groups based on GO terms and KO terms respectively (**Figure S2**). SaVir up-regulated genes appeared to be assigned to “cell cycle and proliferation”, “metabolism” and “genetic information processing” (**Figure 3** **and Figure S2**). SVir up-regulated genes were assigned into groups related to “cellular processes” that included “cell motility and communication”, “cell growth and death” and “cytosqueleton”, to “signal transduction”, and to “response to stimulus” (**Figure 3** **and Figure S2**). Genes without GO terms were annotated manually (using Gene Cards informations) to enrich functional annotations in both DGE libraries.

### Biological functions related to oyster successful response to virulent *Vibrio* infections

We focused on further identification of biological functions that may influence the successful immune response to virulent vibrios. Considering their occurrence in SVir *versus* SaVir DGE library, a list of genes has been established that highlights different pathogen-responsive immune functions (**Table S1**). This list has been generated from the analysis of full data provided in Supplementary file giving information on DGE tag sequences, *C. gigas* EST names, and on Blast2GO and KEGG functionnal annotation (**Table S2**). Several immune-related genes already characterized in oyster have been evidenced with no differential representation between the SVir and SaVir libraries (**Table S2**).

***Immune response*** is characterized by genes related to signaling pathways notably involved in innate immunity (Toll-like/NF-κB and MAPK), from membrane receptors (Toll-like receptor) to regulating intermediaries (MAP kinases, G-protein, and NF-κB inhibitor) and transcription factors (LITAF-like protein). In addition, signaling and interaction molecules such as cytokine receptors were evidenced as well as recognition molecules such as lectins. Besides, proteases, protease inhibitors and stress proteins (such as heat shock proteins and metallothionein) were found with various immune effectors such as antimicrobial (lysozyme). ***Cell adhesion and communication*** category is dominated in SVir by regulating elements associated to cellular processes represented by genes related to cell membrane molecules like integrins, collagen or tetraspanins. ***Cytosqueleton reorganisation*** appears as a dominant group in terms of number of sequences associated to cellular and response processes related to actin (calcium dependent) and tubulin reorganization. Particularly, actin related genes associated to endocytosis were evidenced such as genes coding for membrane receptors associated to vesicule formation or to endosome and lysosome. In addition, trafficking and autophagy are highlighted by genes encoding several trafficking proteins and regulators of transport associated to cytosqueleton network. ***Respiratory chain***, dominated by regulating and response elements, is represented by genes associated to oxidative stress, several potent antioxidants and potential DNA repair elements associated to respiratory burst. ***Apoptosis*** is noticed with both regulating pro-apoptotic related genes such as caspases but also anti-apoptotic ones, such as Baculoviral IAP repeat-containing proteins, involved in inhibition of tumor necrosis factor receptor-associated factors. Finally, ***Cellular differentiation and proliferation*** category is represented by regulating and response elements like genes involved in TGF-β and Wnt signaling pathways and positive or negative regulators of these pathways (such as small GTPase).

## Discussion

Here, we report on the first deep sequencing study of the transcriptome of hemocytes, immunocompetent cells, of the oyster *Crassostrea gigas*. We have identified by DGE approach those defence mechanisms related to the oyster successful response and survival to virulent *Vibrios* comparatively to non virulent *Vibrio* infection. Only the animals that were able to survive infections were considered for the DGE library construction, *i.e.* hemocytes were collected after the peak of mortalities, 96 h post-infections. To fulfill this requirement, oysters have been experimentally infected by injection of the bacteria into the adductor muscle, since this method is the only reproducible and standardized procedure to induce mortality of oysters with controlled doses of virulent *Vibrio* [24]. Comparatively, the immersion in virulent *Vibrio*-containing sea water does not consistently induce disease with well defined peaks of mortalities. It is noteworthy that, in our experimental conditions, bacteria virulence mechanisms that can breach the first line of oyster defences have been bypassed. Besides, potential recognition processes have not been highlighted in the analyses. Thus, the present study is not exhaustive. By considering surviving oysters after the peak of mortalities, we have focused on late processes of the immune responses relevant to bacteria elimination and infection resolution, instead of defence mechanisms occurring early post-infection.

Functional annotation of genes differentially represented between the SVir and SaVir DGE libraries has revealed biological processes that may characterize the successful immune response of oysters to pathogenic vibrios *versus* a non pathogenic one. However, because *C. gigas* is a non-model organism, it is noteworthy that such annotations may only suggest putative functions. The response to avirulent *Vibrio* was enriched in genes related to cell metabolism and cell cycle which may reflect hemocyte homeostasis recovery upon non-pathogenic challenge. Comparatively, in respect to oyster survival to infections, we found enrichment for genes related to signal transduction, signaling molecules and interactions, implicated in the regulation of immune response, as well as to cell adhesion and communication. In addition, the hemocyte transcript catalog we generated highlights cellular processes and defence mechanisms related to phagocytic events, actin cytosqueleton reorganization, cell trafficking and autophagy, but also to oxidative antioxidant and anti-apoptotic reactions.

The hemocyte gene repertoire of oysters able to circumvent pathogenic infections revealed the presence of **pathogen recognition molecules and elements of signaling pathways** involved in immune responses and inflammatory processes. Two lectins were seen up-regulated in response to virulent vibrios, a homologue to L-rhamnose-binding lectin (RBL) identified as constitutively expressed in *Hydractinia* [25] and the oyster C-type lectin. RBLs are pattern recognition receptors in vertebrates; they have opsonising properties and are activators of pro-inflammatory cytokines [26]. Interestingly, here, the putative RBL was seen to be exclusively expressed in response to the virulent vibrios according to a pathogen-specific expresssion signature for *V. aestuarianus*. C-type lectins are Ca^+^-dependent carbohydrate binding proteins. Upon pathogen recognition, they are known as mediating various immune responses in invertebrates and vertebrates such as autophagy, phagosome maturation or apoptosis [27], [28]. One can notice that in the DGE libraries, oyster galectin, a galactosidase-binding lectin known to be involved in infections [29], was not seen differentially expressed according to the bacterial virulence (**Table S2**). However, in vertebrates, galectins play dual functions as damage-associated molecular patterns (DAMPs) and as receptors for pathogen-associated molecular patterns (PAMPs) [30]. Among pattern recognition receptors (PRRs), a Toll-like receptor homologous to Toll-9 from *Anopheles* was evidenced as differentially expressed, whereas the Toll-like receptor 1, recently described in oyster [31], did not (**Table S2**). Our results enriched the Toll-like repertoire in oyster, but further investigations will determine the respective involvement of these molecules in innate immune pathways, particularly the Toll/NF-κB pathway. Among SVir up-regulated genes, we identified several components of *C. gigas* NF-κB pathway [17] as well as LITAF (LPS-Induced TNF-α Factor) transcription factor already identified in *C. gigas* [32]. LITAF signaling pathway plays a major role in regulating various mouse inflammatory cytokines in response to LPS stimulation [33]. Here, cytokine receptor and cytokine-induced protein categories related to inflammation processes were also evidenced with up-regulated putative tumor necrosis factor ligand superfamily member 10, and Interferon-induced protein 44. Proinflammatory effects of virulent *Vibrio* infections were also revealed by over representation, in oyster SVir library, of components of MAPK signaling pathway. In abalones, pathogenic *V. harveyi* avoids both phagocytosis and ROS production and reduces p38 MAPK [34].

Besides cytokines and associated proteins, **protease inhibitors** were also found. Among them, *Cg*-TIMP, an inhibitor of metalloproteinases, which has NF-κB binding sites in promoting region, was shown to be implicated in oyster wound healing and defence mechanisms [16]. Several putative cystatins A and B and serpins were also identified whose putative role in invertebrate immune defence was suggested by previous genomic studies [35]. Vibrios secrete extracellular metalloproteases whose toxicity has been demonstrated both for *V. splendidus* LGP32 [36] and *V. aestuarianus* strain [37]. Thus, we can hypothesize that such protease inhibitors neutralize the *Vibrio* toxins. Moreover, members of cystatin superfamily have immunomodulatory properties and cytokine regulating properties thus preventing excessive inflammation (for review [38]). Cystatins may also interact with TIMPs and other proteases in patho-physiological processes that require tissue remodeling and that range from cell survival and proliferation, to differentiation and cell signaling [39]. High representation of sequences for stress proteins was shown, including various heat shock proteins (HSPs) and oyster metallothioneins (MTs). MTs are ubiquitous metal binding proteins though to be involved in detoxifying of heavy metals, in free radical scavenging but also in inflammatory responses [40].

Surprisingly, whereas we evidenced the potential involvement of immune response regulating pathways, few antimicrobials were seen to be significantly involved in the response to infections. Thus, no differential representation of antimicrobial transcripts was observed in the DGE data, namely for *Cg*-BPI, Bactericidal/Permeability-Increasing protein [12], *Cg*-Prps, proline-rich peptides [14], or for the new oyster big defensin family (Rosa *et al.* in prep). However, lysozyme transcripts were significantly increased in hemocytes from oyster surviving virulent *Vibrio* infection that motivates further characterization of this antimicrobial. Indeed, until now, three lysozymes have been characterized in oyster, mainly from mantle, gills or digestive gland with potential digestive functions [41]. Finally, one can mention the high representation of transcripts with homologies with Complement component 1q (C1q) in the SVir library. C1q domain containing (C1q-DC) proteins are ubiquitous in animal kingdom and many of them remain to be characterized. Nevertheless, C1q-DC proteins display many functions in immunity including clearance of pathogens in vertebrates [42] and invertebrates as well [43].

The DGE data presented here revealed the importance of genes involved in **cell adhesion and communication** that may contribute to oyster post-infection recovery. Among them, we showed several integrins including an oyster β-integrin that plays an important role in phagocytosis [44]. Integrins are also cell adhesion receptors for proteins of the extracellular proteins such as collagens evidenced here. Collagens maintain tissue integrity but also play significant role in regulating cell functions. Therefore, collagen production may indicate potential repairement of the lesions caused by virulent *Vibrio* infection. Besides, interestingly, members of tetraspanin superfamily were also significantly represented. Tetraspanins are small transmembrane proteins that, once interacting with immune receptors such as integrins, play important role in cellular processes such as migration, proliferation. They are immune modulators of signaling pathways (NF-κB and kinases) that induce cytokine production following pathogenic recognition [45]. Whereas tetraspanin associated to phagocytic receptors contribute to facilitate phagocytosis processes, microbial pathogens can also exploit tetraspanin to enter in host cell for further colonization and invasion [46]. Interestingly, *V. splendidus* LGP32 uses β-integrin through its outer membrane protein OmpU to invade oyster hemocyte and further impair defence fonctions [19]. Whether tetraspanins intervene also in *V. splendidus* LGP32 pathogenesis remains to be established. However, because tetraspanin transcript enrichment was shown here to be associated to the oyster successful response to circumvent *Vibrio* infection, it is likely that they display a role in modulating inflammatory responses. In several mollusk species, pathogenic vibrios (*i.e. V. aestuarianus*) use cytotoxins (metalloproteases and extracellular products - ECPs) as part of their virulence mechanisms by impairing cellular processes including adhesiveness, migration, morphogenesis and phagocytosis [47], [48]. Thus, in our study, the oyster capability to have circumvented the infection is reflected by the enrichment of gene transcripts related to cell adhesion and motility, and consequently to cytosqueleton reorganization.

We evidenced numerous molecules involved in **cytoskeleton rearrangements** which are essential for various cellular processes dealing with morphogenesis, chemotaxis, migration but also phagocytosis and intracellular transports [49]. Thus, actin cytoskeleton changes are also involved in host-pathogen interactions. However, many pathogens subvert these cellular machineries for invading and surviving into host cells [50] as also reported for the oyster pathogen *V. splendidus* LGP32 [19]. Here, the DGE approach greatly contributed to characterize in *C. gigas* numerous genes, actors of cellular defence mechanisms, but consequently also potential targets for *Vibrio* virulence. Among those, one can cite overexpression of genes related to endocytosis, lysosome and active state of actin as well as to tubulin reorganization. All those cellular motor genes (myosin light chain, dynein arm light chain, kinesin-like protein) involved in intracellular trafficking or exocytosis of immune effectors may contribute to removal of invading pathogen and homeostasis recovery. This is particularly highlighted by the modulation of genes we classified in autophagy and microtubule transport. Authophagy is part of the microbicidal defence system. This conserved mechanism plays roles in degrading intracellular pathogens but also in regulator of innate immunity particularly the inflammatory or systemic immune response [51].

Apoptosis, another specialized form of **programmed cell death**, was also evidenced in our data with different pro- or anti-death effectors such as caspases, cytochrome c or IAPs. Baculoviral IAP repeat-containing 2/3/4 is described to inhibit apoptosis by binding to tumor necrosis factor receptor-associated factors [52]. Thus, hemocytes of surviving oysters up-regulate a variety of anti-apoptotic genes which could modulate inflammatory cytokine pathways and oxidative stress potentially toxic for the cell. Besides activation by inflammatory pathways, apoptosis as autophagy can be triggered by **reactive oxygen species** (ROS) [53]. Here, the successful response of oyster to virulent *Vibrio* infection revealed up-regulated genes related to respiratory chain and particularly in ROS production, a microbicidal defence reaction known in oyster [54], as well as putative genes coding for anti-oxidants. In *C. gigas*, hemocyte oxidative metabolism has been shown to be enhanced following virulent *V. aestuarianus* infection when superoxide dismutase *Cg*-EcSOD gene was down-regulated, likely being a pathogen adaption for impairing hemocyte functions and survival [20]. With respect to *V. splendidus* LGP32, we have shown that the *Vibrio* uses *Cg*-EcSOD as opsonin for hemocyte invasion and evades defence reaction by limiting ROS production [19]. Our DGE data provided several components of the oxidative stress that may contribute to pathogen elimination and to anti-inflammatory reactions, in the context of oyster survival to infections.

Our study highlighted signaling pathways and regulators we classified in **cell differentiation**, closely related to the different functions described above. Those are homologous to activins and TGF-β inducible early growth response proteins, members of the TGF-β signaling pathway which regulates a wide spectrum of cellular functions such as proliferation, apoptosis, differentiation and migration [55]. Additionally, the involvement of Wnt-signaling pathway and numerous members of the Rho protein family such as Cdc42 and small GTPases were evidenced. They are important actors for cellular processes such as migration, chemiotaxis or phagocytosis and they are involved in cellular functions related to actin cytoskeleton regulation. Thus, Rho GTPases are also known to be specific targets for bacterial cytotoxins [56].

Here, qPCR analyses aimed at validating the differential gene expression evidenced by DGE. So, independent experimental infections were performed separately with the different virulent and avirulent vibrios. Using oysters from the Mediterranean sea instead of the Atlantic ocean, we showed that the differential gene expression was independent of the oyster origin. Interestingly, pathogen-specific signatures were evidenced upon bacterial challenge. To our knowledge, this is shown for the first time in this invertebrate. The different oyster infections resulted in both responses shared by the three *Vibrio* strains whatever their virulence or avirulence, and specific responses to virulent vibrios. This is consistent with recent data on virulence mechanisms and pathogenesis that greatly differ between *V. aestuarianus* strain and *V. splendidus* LGP32. *V. aestuarianus* was shown to affect hemocyte phagocytosis and adhesiveness properties by the secretion of extracellular products and to enhance the production of ROS [48]. Regarding *V. splendidus* LGP32, a metalloprotease from ECPs (extracellular products) has been associated to toxicity and its outer membrane protein OmpU has been evidenced as a virulent factor [24], [36]. Recently, *V. splendidus* LGP32 has been shown to be a facultative intracellular pathogen which invades the oyster hemocytes through OmpU adhesin/β-integrin recognition and survives by impairing phagosome acidification and ROS production [19]. In this work, we demonstrated that bacterial invasion induces hemocyte cytoskeleton reorganizations by analysing expression of genes evidenced in the present DGE study.

### Concluding remarks

This genome-wide expression profiling aimed at a better understanding of the molecular mechanisms that control or contribute to the anti-infectious response of this marine bivalve mollusc with economical importance. The data we generated for characterizing the transcriptome of the oyster immune functions could be enriched by further progress on genomic resources of this non-model organism. Nevertheless, we provided here a catalog of genes and cellular or immune functions that are potential targets for mechanisms of pathogen resistance and escape to the immune response, that may concern not only *Vibrio* species but also viruses. Further exploitation of our DGE libraries opens the way to (i) the in-depth characterization of pathogen-specific gene expression signatures and (ii) the description of the effects of vibrio/virus co-infections that may impair oyster immune defences. Indeed, it is now considered that Herpes virus OsHV-1 together with *Vibrio* species, such as *V. splendidus* LGP32 present in the oyster microbiota, may contribute to *C. gigas* mortality outbreaks. Finally, this work is a prerequisite for the identification of those physiological traits controlling oyster survival capacity and subsequently for a better understanding of the phenomenon of summer mortality.

## Materials and Methods

### Bacterial strains

Two strains belonging to the *Vibrio splendidus* polyphyletic group were considered, namely the oyster pathogen *V. splendidus* LGP32 [4] and *V. tasmaniensis* LMG 20012^T^ used as an avirulent strain [57]. Additionally, virulent *V. aestuarianus* LPi 02/41 isolated during oyster mortality events was chosen as representative of this dominant *Vibrio* species [3]. The strains were grown under agitation at 20°C in artificial sea water (ASW) [58] supplemented with 4 g/l bactopeptone and 1 g/l yeast extract (referred to as Zobell medium) for 18 h. Bacterial concentrations were evaluated by optical density (OD) at 600 nm (UltraspecIII, Pharmacia Biotech), an OD value value of 1 corresponding to 10^9^ colonies forming units (CFU)/ml for *V. aestuarianus* LPi 02/41 and to 2.10^9^ for *V. splendidus* LGP32 and *V. tasmaniensis* LMG 20012^T^. Bacteria were centrifuged (15 min, 3,000×g, 20°C) and suspended in autoclaved ASW at the concentration calculated for the experimental injections.

### Oysters and experimental infections

For DGE library construction, adult (2 year-old) oysters, *Crassostrea gigas*, were purchased from an Atlantic oyster farm (La Tremblade, France) and acclimatized in the Ifremer laboratory (LGP, La Tremblade, France) over a 1-week period in aerated 0.45 µm-filtered seawater. The temperature was maintained at 20°C during the trial. A total of 390 oysters was individually tagged and distributed in three groups in separate tanks. For infection, oysters were first anesthetized for 3 h in aerated 50 g/l MgCl_2_ bath (2/3 v/v sea water/freshwater) containing phytoplankton (*Chaetoceros gracilis* and *Isochrysis galbana*). Experimental infections were performed as previously described by injecting bacteria into the posterior adductor muscle [24]. One group of 180 oysters was injected with 8.10^7^ CFU of *V. splendidus* LGP32 per animal under 100 µl, and a second group (180 osyters) with 2.10^7^ CFU/animal of *V. aestuarianus* LPi 01/42. The third group (30 oysters) was injected with 8.10^7^ CFU/animal of the avirulent *Vibrio tasmaniensis* LMG 20012^T^.

A second experimental infection was performed for qPCR analyses with adult oysters (2 year-old) obtained from a Mediterranean commercial hatchery (Sodimer, Montpellier, France). Oysters were acclimatized at 20°C and maintained in tanks with UV-treated and biologically filtered sea water in the experimental aquaculture platform of Ifremer Palavas (France). To allow the intramuscular injection of bacteria suspensions, a small cut was made in the side of oyster shells, adjacent to the adductor muscle. A total of 300 oysters were divided into four groups. Two groups of 100 oysters were respectively infected by virulent *Vibrio* strains, 4.10^8^ CFU/animal of *V. splendidus* LGP32 and 2.10^7^ CFU/animal of *V. aestuarianus* LPi 02/41. The third group of 50 oysters was injected with 2.10^8^ CFU/animal of the avirulent strain *V. tasmaniensis* LMG 20012^T^. Finally, the fourth group of 50 non infected oysters was used as control for the experiment to assess mortality due to the handling of the animals.

For both experiments, mortalities were monitored daily and, at the end of mortalities, hemolymph was individually collected from surviving oysters by withdrawing 0.5 to 1 ml from the posterior muscle adductor using a precooled 2 ml syringe for further RNA extraction. The non-parametric method of Kaplan-Meier (XLSTAT 2008.7.02) test was used to estimate survival rates and the Log-Rank and Wilcoxon values for comparing differences between the groups. All experimental infections were performed according to the Ifremer animal care guideline and policy.

### RNA extraction

Hemocyte samples from individual oysters were obtained by hemolymph centrifugation at 1,500×g for 15 min at 4°C. Each pellet was lysed in 1 ml of TRIzol® reagent (Invitrogen®) for total RNA extraction according to the manufacturer's instructions. Total RNA amount and purity were checked by using spectrophotometrer NanoDrop ND-1000 (Thermo Scientific, Les Ulis, France) and the integrity of total RNA was analyzed by agarose-electrophoresis.

### DGE library construction and sequencing

Two DGE libraries were constructed from hemocyte total RNA of oysters surviving *Vibrio* infections: SVir from pooled RNA samples from surviving individuals of virulent infections with *V. splendidus* LGP32 and *V. aestuarianus* LPi 02/41; SaVir from individuals injected with the avirulent *V. tasmaniensis* LMG 20012^T^.

Sequence tag preparation was done with Illumina's Digital Gene Expression Tag Profiling Kit according to the manufacturer's protocol (version 2.1B). For both libraries, 7 µg of total RNA (from 10 oysters, 0.7 µg per oyster) was incubated with oligo-dT beads. First- and second-strand cDNA syntheses were performed using superscript II reverse transcription kit according to the manufacturer's instructions (Invitrogen). The cDNAs were cleaved using the *NlaIII* anchoring enzyme. Subsequently, digested cDNAs were ligated with the GEX adapter 1 containing a restriction site of *MmEI*. The second digestion with *MmeI* was performed, which cuts 17 bp downstream of the CATG site. At this point, the fragments detach from the beads. The GEX adapter 2 was ligated to the 3' end of the tag. A PCR amplification with 15 cycles using Phusion polymerase (Finnzymes) was performed with primers complementary to the adapter sequences to enrich the samples for the desired fragments. The resulting fragments of 85 bp were purified by excision from a 6% polyacrylamide TBE gel. The DNA was eluted from the gel debris with 1× NEBuffer 2 by gentle rotation for 2 h at room temperature. Gel debris were removed using Spin-X Cellulose Acetate Filter (2 ml, 0.45 µm) and the DNA was precipitated by adding 10 µl of 3 M sodium acetate (pH 5.2) and 325 µl of cold ethanol, followed by centrifugation at 13,000×g for 20 min. After washing the pellet with 70% ethanol, the DNA was resuspended in 10 µl of 10 mM Tris-HCl (pH 8.5) and quantified by using Nanodrop 1000 spectrophotometer.

Cluster generation was performed after applying 4 pM of each sample to the individual lanes of the Illumina 1G flowcell. After hybridization of the sequencing primer to the single-stranded products, 18 cycles of base incorporation were carried out on the 1G analyzer according to the manufacturer's instructions. Image analysis and base calling were performed using the Illumina Pipeline, where sequence tags were obtained after purity filtering. This was followed by sorting and counting the unique tags.

### DGE library characteristics and functional annotation of tags differentially represented between libraries

The sequence files of each DGE library were analyzed with BIOTAG software (Skuldtech, Montpellier, France). The statistical value of DGE data comparisons, as a function of tag counts, was calculated by assuming that each tag has an equal chance of being detected. For several highly expressed transcripts, we checked that tag frequencies in successive sequence batches were distributed in agreement with a binomial law [59]. Selected genes were chosen based on a comparison between the two libraries, combined with the significance threshold of the observed variations (*p-value*<0.01). Tag to gene mapping was performed using EST collection from *C. gigas* which contains 29,745 unique sequences (7,940 contigs and 21,805 singletons), generated from different oyster tissues including hemocytes, and stored on the platform of Sigenae-INRA Toulouse (http://www.sigenae.org/aquafirst/). For tag to gene mapping, the virtual tags were extracted from all contigs and singletons.

Sequence functional annotation analyses were performed using two ways of classification. First we used Blast2GO software v1.3.3. (http://www.blast2go.org/start_blast2go). Briefly, Blast2GO uses BlastX available through the National Center for Biotechnology Information (NCBI) with a user-defined threshold to find similar sequences from the NCBI (nr database). Sequences which found homology with annotated sequences were annotated according to the gene ontology (GO) terms. The hierarchical representation of the gene ontology is structured according to different levels, from the top (level 1) parents corresponding to the three main GO categories (cellular component, biological process and molecular function) to the lowest more specialized child terms level 2, 3, 4 etc. In the present research, GO annotations were represented at level 3 of biological process. Due to the redundancy of term attribution, we have manually condensed group functionally related gene and term into seven functional groups to clarify annotation. Second, we have used the KEGG Automatic Annotation Server (Kyoto encyclopedia of genes and genomes, http://www.genome.jp/tools/kaas/) with SBH method for EST annotation. This server provides functional annotation of genes by BlastX comparisons against the manually curated KEGG GENES database, for ortholog assignment and pathway mapping. In addition, we have used GenesCards version 3 to obtain functionnal description of indentified genes (http://www.genecards.org/).

### Real-time quantitative PCR analysis

To validate the quantitative data of DGE libraries, we have quantified the expression levels of 35 selected genes from SVir or SaVir DGE libraries by quantitative PCR (qPCR) from pooled hemocyte total RNAs in biological replicates (10 oysters per replicata) from four experimental conditions: (i) uninfected oysters, (ii) oysters injected with avirulent *V. tasmaniensis* LMG 20012^T^, (iii) oysters that survived infection with virulent *V. splendidus* LGP32 or (iv) *V. aestuarianus* LPi 02/41. These experimental conditions were analysed in biological replicates of ten oysters per replicata. The first cDNA strand was synthesized from 700 ng of purified total RNA from hemocytes, using MMLV Reverse Transcriptase kit, according to the manufacturer's instructions (Invitrogen®), in 20 µl of reaction volume. qPCR amplifications were performed in the LightCycler 480 (Roche) in a final volume of 5 µl containing 5 mM MgCl_2_, 0.33 µM of each primer, 2.5 µl of reaction mix (LightCycler 480 SYBR Green I Master 2X) and 1 µl of each reverse transcribed RNA (diluted 1∶9). The list of oligonucleotide primers used to amplify target genes is shown in (**Table S3**). Each qPCR reaction was performed with an initial denaturation step of 10 min at 95°C followed by an amplification of the target cDNA for 40 cycles, each cycle consisting of a denaturation at 95°C for 10 s, annealing at 57°C for 20 s and elongation at 72°C for 25 s. Specificity of the qPCR product was analyzed by melting curve analysis. To determine the qPCR efficiency of each primer pair used, standard curves were generated using six serial dilutions (1∶1, 1∶3, 1∶7, 1∶15, 1∶31, 1∶63) of a unique cDNA sample constituted from a pool of all cDNAs obtained from each condition; qPCR efficiencies of tested genes varied between 1.87 and 1.99. Results are shown as changes in relative expression normalized with the elongation factor 1-alpha reference gene (EF-1α, GenBank accession number **AB122066**) using the 2^−(ΔΔCt)^ method described by Pfaffl [60]. Global hierarchical clustering of qPCR data was performed with Multiple Array Viewer software (version 4.6.2, http://www.tm4.org/mev/) using avarage linkage clustering with Pearson correlation as the default distance metric. In addition, statitical analyses gene by gene were conducted using Statistica Statsoft software version 6 with the non-parametric multiple comparison test ANOVA of Kruskal-Wallis were considered significantly different at *p*<0.05.

## Supporting Information

Table S1 List of functional groups and related hemocyte up-regulated genes from oysters surviving virulent *versus* avirulent *Vibrio* infections.
(PDF)

Table S2 List of annotated genes from hemocyte DGE libraries used for functional annotations.
(XLSX)

Table S3 List of primers.
(XLSX)

Figure S1 ***Hierarchical clustering*** analysis and differential expression of 17 genes from SaVir library. Hemocyte gene expression profiles were analysed in biological replicates from oysters: non injected as control (C), surviving infections with avirulent *V. tasmaniensis* LMG 20012^T^ (T), with virulent *V. splendidus* LGP32 (S) and virulent *V. aestuarianus* LPi 02/41 (A). Each cell in the matrix corresponds to the expression level of one gene in a sample. The intensity of the color from green to red indicates the magnitude of differential expression (see color scale at the bottom of the image). Relative expressions were calculated according the 2^−(ΔΔCt)^ method normalized with elongation factor-1α (EF-1α). Each value was calculated in reference to the mean of ΔCt of all conditions (relative expression = 1). The dendrograms at the top of the figures indicate relationship among experimental conditions which define clusters of conditions (CC). The dendrograms at the left of the figures indicate relationship among the profiles of the selected genes which define clusters of expression (CE), after clustering analysis using Multiple Array Viewer software.
(TIF)

Figure S2 Gene ontology assignment of 1,610 and 2,321 unique ESTs differentially represented between SVir and SaVir libraries, respectively. (a) Categorization based on GO terms assignment from 3^nd^ level of Biological Process using Blast2GO software. (b) Categorization based on KO terms (level A and B) of the Kyoto Encyclopedia of Genes and Genomes (KEGG) using KEGG Automatic Annotation Server (KAAS).
(TIF)
