# Supplementary material for: Whole Transcriptome Profiling of Successful Immune Response to Vibrio Infections in the Oyster Crassostrea gigas by Digital Gene Expression Analysis
Source: PLoS One. 2011 Aug 4;6(8):e23142. doi: 10.1371/journal.pone.0023142 (PMC3150398; doi:10.1371/journal.pone.0023142)
Supplement: Table S1 — List of functional groups and related hemocyte up-regulated genes from oysters surviving virulent versus avirulent Vibrio infections. (PDF) [file pone.0023142.s001.pdf]

Table S1: List of functional groups and related hemocyte up-regulated genes from oysters surviving virulent *versus* avirulent *Vibrio* infections.

Function	Gene name	Species: GenBank accession number	Blastx e-value (identities)	occurrence		No of tags	No of ESTs
				SVir	SaVir		
**Immune response**							
	**Signaling pathways**						
	Toll9	*Anopheles gambiae*: AAL37903	1e-15 (33%)	8	1	1	1
	TGF beta-activated protein kinase kinase kinase 7	*Salmo salar*: ACN10470	4e-24 (52%)	11	5	1	1
	MAP kinase-interacting serine/threonine-protein kinase 1	*Mus musculus*: NP_067436	5e-116 (61%)	71	28	2	1
	Inhibitor of nuclear factor kappa-B kinase subunit epsilon	*Mus musculus*: BAA85154	3e-88 (56%)	3	0	1	1
	Inhibitor kappa B	*Argopecten irradians*: ACZ34178	7e-46 (40%)	10	2	1	1
	Syntenin-1	*Rattus norvegicus*: NP_114192	2e-84 (56%)	89	24	1	1
	Guanine nucleotide binding protein (G protein), gamma 7	*Xenopus tropicalis*: NP_001017133	4e-06 (43%)	13	4	3	3
	Lipopolysaccharide induced TNF-alpha factor	*Chlamys farreri*: ABI79459	1e-35 (86%)	177	74	2	1
	**Cytokine-cytokine receptor interaction**						
	Tumor necrosis factor ligand superfamily member 10	*Sus scrofa*: NP_001019867	1e-09 (26%)	16	2	1	1
	C3a anaphylatoxin chemotactic receptor	*Danio rerio*: P0C7U4	5e-06 (26%)	26	13	1	1
	* Interferon-induced protein 44	*Salmo salar*: NP_001133872	1e-23 (35%)	3	1	1	1
	**Lectin**						
	* C-type lectin 2 like protein	*Crassostrea gigas*: CAE18170	0.0 (100%)	30	7	2	3
	* Rhamnospondin 1, L-rhamnose-binding lectin	*Hydractinia symbiolongicarpus*: ABD95939	7e-17 (44%)	128	0	1	1
	**Protease inhibitor, stress response protein and others**						
	* Cystatin A2	*Dictyostelium discoideum*: XP_641175	4e-13 (52%)	20	9	1	1
	* Cystatin B-like protein	*Crassostrea gigas*: ADI33157	0.0 (100%)	108	43	2	2
	Proteinase inhibitor I4 serpin	*Chlorobium limicola*: YP_001943402	1e-15 (59%)	29	10	2	2
	Tissue inhibitor of metalloproteinase (*Cg*-TIMP)	*Crassostrea gigas*: AAT73610 and AAG42824	0.0 (100%)	5727	2640	4	5
	Heat shock protein 70	*Crassostrea gigas*: AAD31042	0.0 (100%)	140	67	1	1
	* Heat shock protein 22 isoform 1	*Venerupis philippinarum*: ACU83231	3e-22 (61%)	158	40	2	4
	Heat shock protein beta-1	*Caligus clemensi*: ACO14969	3e-12 (40%)	110	24	1	1
	Small heat shock protein	*Trichinella pseudospiralis*: ABJ55915	2e-16 (37)	5	0	1	1

	Universal stress protein G	*Schistosoma mansoni*: XP_002578809	2e-14 (32%)	13	5	1	1
	Serum amyloid protein A	*Branchiostoma belcheri*: BAB97379	8e-45 (83%)	59	28	1	1
	Viperine-like protein	*Suberites domuncula*: CAL36990	2e-79 (70%)	3	1	1	1
	Lysozyme 1	*Crassostrea gigas*: BAD19059	0.0 (100%)	254	82	1	1
	Complement component 1q subcomponent-like 1-like	*Danio rerio*: XP_695928	1e-4 (46%)	40	1	1	1
	* Metallothionein IV	*Crassostrea gigas*: CAK22381	0.0 (100%)	15095	6212	7	6
**cell adhesion-communication**							
	* Integrin beta-PS	*Camponotus floridanus*: EFN70798	1e-15 (35%)	24	7	1	1
	Cysteine and histidine-rich domain-containing protein 1	*Gallus gallus*: NP_001026003	7e-71 (57%)	8	1	1	1
	Calcium and integrin-binding protein 1	*Camponotus floridanus*: EFN62878	8e-50 (64%)	35	14	2	2
	Collagen alpha-1(XII) chain precursor	*Gallus gallus*: NP_99035	4e-16 (40%)	3	0	1	1
	Collagen, type XXII, alpha 1	*Gallus gallus*: NP_001132911	5e-23 (40%)	4	0	1	1
	Collagen, type X, alpha 1	*Danio rerio*: NP_001077296	9e-09 (33%)	5	1	1	1
	Fibrillar collagen	*Arenicola marina*: AAC47545	2e-07 (69%)	10	2	1	1
	Collagen receptor related protein	*Crassostrea gigas*: AAQ13478	0.0 (100%)	20	6	1	1
	Collagen pro alpha-chain	*Haliotis discus*: BAA75668	4e-40 (41%)	8	2	1	1
	Collagen protein	*Mytilus edulis*: ABA03049	3e-29 (52%)	11	1	1	1
	Procollagen	*Loa loa*: XP_003139504	2e-04 (75%)	346	117	1	1
	Neural-cadherin	*Camponotus floridanus*: EFN64630	2e-14 (45%)	32	10	2	1
	Cytohesin-1	*Harpegnathos saltator*: EFN84340	8e-10 (76%)	2	0	1	1
	Dermatopontin 2	*Haliotis diversicolor supertexta*: ACH48240	1e-32 (46%)	39	8	1	1
	Tetraspanin	*Heliocidaris tuberculata*: ABE27955	7e-17 (29%)	358	0	1	1
	Tetraspanin 33	*Danio rerio*: CAI20758	5e-11 (37%)	67	0	1	1
	Tetraspanin 96F	*Drosophila melanogaster*: NP_524500	5e-09 (33%)	44	20	1	1
	Protein kish-A precursor	*Gallus gallus*: NP_001106925	1e-17 (83%)	4	2	1	2
	Transmembrane protein 79, isoform CRA_a	*Homo sapiens*: EAW52970	2e-06 (25%)	5	1	1	1
	Dermal papilla derived protein, putative	*Ixodes scapularis*: XP_002399750	8e-36 (37%)	6	3	1	1
	Transmembrane protease, serine 2	*Xenopus laevis*: NP_001090208	1e-34 (48%)	8	2	1	1
	Fibrillin-2	*Harpegnathos saltator*: EFN80554	3e-120 (39%)	16	8	1	1
	Adhesion molecule homolog family member (lad-2)	*Caenorhabditis elegans*: NP_001023497	3e-09 (32%)	4	2	1	1
	Flamingo 1	*Gallus gallus*: AAR83924	3e-13 (42%)	14	4	1	1
	Gamma-secretase subunit pen-2, putative	*Pediculus humanus corporis*: XP_002426899	3e-29 (65%)	4	0	1	1

	Osteonectin-like protein	*Phragmatopoma lapidosa*: ACV72069	7e-52 (46%)	3	0	1	1
**Cytosqueleton reorganisation**							
	**Actin related molecules**						
	Actin 2	*Crassostrea gigas*: BAB84579	0.0 (100%)	2546	799	2	1
	Ankyrin repeat protein	*Trichomonas vaginalis*: XP_001314547	4e-39 (42%)	3	0	1	1
	* Radixin	*Mus musculus*: EDL25785	1e-94 (88%)	3	1	1	1
	F-actin-capping protein subunit alpha	*Harpegnathos saltator*: EFN89112	4e-107 (67%)	4	2	1	1
	Kelch-like protein 5	*Danio rerio*: NP_001164413	7e-17 (73%)	19	6	1	1
	Ring canal kelch protein	*Culex quinquefasciatu*: XP_001843614	2e-17 (24%)	36	6	1	1
	Actin-interacting protein 1	*Trichinella spirali*: EFV49821	7e-26 (40%)	39	16	1	1
	Myosin light polypeptide 6	*Lycosa singoriensis*: ABX75511	2e-52 (68%)	128	39	1	2
	Myosin essential light chain	*Crassostrea gigas*: CAD91423	0.0 (100%)	885	118	5	1
	Myosin heavy chain cardiac muscle specific isoform 2	*Argopecten irradians*: AAF62395	5e-05 (72%)	102	22	1	1
	Myosin heavy chain	*Placopecten magellanicus*: AAB03661	5e-42 (81%)	30	11	1	1
	Myosin II heavy chain	*Polysphondylium pallidum*: EFA84750	2e-06 (24%)	5	0	1	1
	Myosin regulatory light chain	*Pecten maximus*: AAD52844	3e-18 (63%)	9	0	1	1
	Myosin regulatory light chain	*Placopecten magellanicus*: AAB02931	5e-59 (71%)	28	10	1	1
	Titin	*Harpegnathos saltator*: EFN83272	5e-28 (29%)	8	3	1	1
	Titin	*Mus musculus*: EDL27217	1e-42 (37%)	11	0	1	1
	Paramyosin protein	*Crassostrea gigas*: CAD79338	0.0 (100%)	4	2	1	1
	Protein Shroom2	*Xenopus tropicalis*: NP_001072178	1e-21 (47%)	5	1	1	1
	Receptor of activated kinase C	*Crassostrea angulata*: ACU33969	0.0 (100%)	4	0	1	1
	* Fascin	*Parasteatoda tepidariorum*: BAI83409	2e-27 (41%)	15	2	1	1
	Partitioning defective protein 6	*Trichinella spiralis*: EFV60907	9e-22 (60%)	13	5	1	1
	Cortactin isoform a variant	*Homo sapiens*: BAD96333	2e-09 (76%)	7	3	1	1
	Calponin-like protein	*Mytilus galloprovincialis*: BAB60813	5e-105 (54%)	8	2	2	2
	Calcineurin B subunit	*Pinctada fucata*: ACI96107	2e-83 (98%)	18	2	2	1
	**Endocytose related molecule**						
	Low-density lipoprotein Receptor Related family member (lrp-1)	*Caenorhabditis elegans*: NP_492127	5e-09 (38%)	7	3	1	1
	Tyrosine phosphatase	*Crassostrea gigas*: ACH42087	0.0 (100%)	9	1	1	1
	Vacuolar protein sorting-associated protein 28-like protein	*Harpegnathos saltator*: EFN81106	4e-79 (73%)	32	6	1	1
	AP-2 complex subunit beta-1, putative	*Ixodes scapularis*: XP_002412040	5e-28 (70%)	124	49	1	1

	Charged multivesicular body protein 2a	*Xenopus tropicalis*: NP_001005008	3e-46 (84%)	3	1	1	1
	Charged multivesicular body protein 2b	*Ictalurus punctatus*: NP_001187612	4e-26 (60%)	4	2	1	2
	Myoferlin	*Rattus norvegicus*: NP_001178565	5e-10 (42%)	26	6	1	1
	**Lysosome**						
	Lysosome-associated membrane glycoprotein 1	*Harpegnathos saltator*: EFN79114	6e-15 (56%)	6	0	1	1
	Lysosomal thioesterase PPT2	*Danio rerio*: NP_001103329	2e-57 (53%)	25	1	3	1
	Hexosaminidase A	*Danio rerio*: NP_001108317	1e-12 (30%)	40	17	1	1
	Stearyl-CoA desaturase	*Cyprinus carpio*: AAB03857	2e-67 (61)	6	3	1	1
	Ganglioside GM2 activator precursor	*Euprymna scolopes*: AAN35173	3e-24 (33%)	33	11	4	4
	Vacuolar H+ ATPase	*Ixodes scapularis*: XP_002433961	4e-53 (87%)	34	15	2	2
	**Tubulin related molecules**						
	Alpha tubulin-like	*Saccoglossus kowalevskii*: NP_001171794	4e-72 (92%)	6	1	1	1
	Tubulin, beta 2C	*Homo sapiens*: AAH24038	8e-06 (97%)	134	62	1	1
	Axonemal dynein light chain p33	*Haliotis discus discus*: ABO26694	1e-118 (93%)	16	7	1	1
	Dynactin subunit 6	*Danio rerio*: NP_001018487	7e-57 (61%)	9	4	1	1
	Kinesin-like protein KIF9	*Rattus norvegicus*: NP_001178929	2e-21 (43%)	7	3	1	1
	Kinesin-II 95 kDa subunit	*Strongylocentrotus purpuratus*: NP_999817	3e-24 (55%)	4	2	1	1
	Growth arrest-specific 8	*Xenopus laevis*: NP_001090062	7e-23 (82%)	10	4	1	1
	**Autophagy and microtubule transport**						
	Niemann-pick C1	*Aedes aegypti*: XP_001656693	1e-72 (56%)	8	3	1	1
	Copper-transporting ATPase 1	*Camponotus floridanus*: EFN70280	5e-71 (65%)	5	1	1	1
	Non-specific lipid-transfer protein	*Danio rerio*: NP_957159	0.0 (68%)	6	0	1	1
	Translocon-associated protein subunit alpha	*Harpegnathos saltator*: EFN87563	1e-47 (58%)	14	7	1	1
	Signal recognition particle, subunit Srp9, putative	*Ixodes scapularis*: XP_002411259	6e-28 (78%)	3	1	1	1
	Protein translocation complex beta subunit, putative	*Aedes aegypti*: XP_001657235	4e-18 (62%)	8	4	1	1
	Major vault protein	*Strongylocentrotus purpuratus*: NP_001116989	2e-48 (74%)	5	1	1	1
	Syntaxin 1A	*Lymnaea stagnalis*: AAO83845	2e-123 (93%)	4	0	1	1
	Mitochondrial import receptor subunit TOM20 homolog			10	1	1	1
	Mitochondrial import inner membrane translocase, subunit TIM23, putative	*Ixodes scapularis*: XP_002433737	2e-19 (37%)	4	2	1	1
	Mitochondria associated granulocyte macrophage csf signaling molecule	*Aedes aegypti*: XP_001662322	6e-23 (59%)	4	0	1	1
	Purinergic ATP P2X4 receptor subunit 1	*Aplysia californica*: AAR28669.	2e-74 (52%)	10	4	1	2
	Gamma-aminobutyric acid receptor-associated protein-like 2	*Osmerus mordax*: ACO09769	2e-41 (77%)	18	3	2	2

	GABA(A) receptor-associated protein	*Haliotis diversicolor*: ADI56518	9e-57 (98%)	11	2	1	1
	Serine/threonine-protein kinase	*Crassostrea ariakensis*: ABY48704	4e-40 (99%)	13	5	1	1
	Autophagy-specific gene 4	*Nasonia vitripennis*: NP_001123274	7e-44 (49%)	3	0	1	1
	Beclin-1-like protein	*Camponotus floridanus*: EFN67583	3e-16 (91%)	8	3	1	1
**Respiratory chain**							
	**Oxydative stress**						
	Reactive oxygen species modulator 1	*Xenopus tropicalis*: NP_001165117	4e-21 (65%)	4	2	1	1
	Mitochondrial F1F0-ATP synthase subunit epsilon/ATP15	*Glossina morsitans morsitans*: ADD20505	7e-11 (64%)	4	2	1	1
	Cytochrome c oxidase subunit II	*Crassostrea gigas*: NP_037546	0.0 (100%)	9	2	1	1
	Cytochrome c oxidase subunit Vb/COX4	*Simulium nigrimanum*: ACZ28304	2e-17 (52%)	38	19	1	1
	Cytochrome b	*Crassostrea gigas*: ACD35414	0.0 (100%)	74	33	2	2
	Succinate dehydrogenase cytochrome B small subunit precursor	*Branchiostoma belcheri tsingtauense*: AAQ18144	4e-18 (49%)	58	19	1	1
	NADH dehydrogenase [ubiquinone] iron-sulfur protein 8, mitochondrial precursor	*Pongo abelii*: NP_001125353	2e-79 (74%)	88	10	1	1
	NADH dehydrogenase 1 alpha subcomplex subunit 6	*Epinephelus coioides*: ADG29146	8e-32 (51%)	7	2	1	1
	NADH dehydrogenase [ubiquinone] 1 beta subcomplex subunit 7	*Trichinella spiralis*: EFV51952	4e-21 (47%)	5	1	1	1
	Selenoprotein W, 1	*Danio rerio*: AAH92686	4e-27 (69%)	81	39	1	1
	Alternative oxidase	*Crassostrea gigas*: ACL31211	0.0 (100%)	112	27	2	1
	Hexokinase	*Crassostrea gigas*: CAJ28915	0.0 (100%)	112	49	2	2
	**Anti-oxidant**						
	* Microsomal glutathione S-transferase	*Venerupis philippinarum*: ACU83223	4e-30 (50%)	85	1	1	1
	Glutathione S-transferase theta	*Neanthes succinea*: ABQ82132	2e-44 (51%)	18	0	1	1
	Omega class glutathione S-transferase	*Crassostrea gigas*: CAD89618	0.0 (100%)	4	2	1	1
	Superoxide dismutase (*Cg*-EcSOD)	*Crassostrea gigas*: AAY60161	0.0 (100%)	24	12	2	1
	DJ-1 (mammalian transcriptional regulator) Related family member	*Caenorhabditis elegans*: NP_493696	1e-50 (60%)	4	2	1	1
	* Nucleoredoxin	*Amphidinium carterae*: ACF28636	2e-29 (51%)	12	3	1	1
	Antioxidant enzyme	*Culex quinquefasciatus*: XP_001865898	1e-14 (55%)	96	23	1	1
	Selenoprotein K	*Salmo salar*: NP_001134373	3e-05 (56%)	10	3	1	1
	Serine/threonine-protein kinase PINK1, putative	*Pediculus humanus corporis*: XP_002432225	6e-27 (35%)	1636	750	1	1
	**DNA repair**						
	alkB, alkylation repair homolog 3	*Xenopus laevis*: NP_001088803	3e-84 (31%)	5	0	1	1
	RAD54 homolog B	*Bos taurus*: NP_001179884		6	3	1	1
	Topoisomerase (DNA) I, isoform CRA_c	*Homo sapiens*: EAW75997	2e-20 (42%)	20	6	1	1

	Retinoblastoma-binding protein 4 (rbbp4)	*Aedes aegypti*: XP_001654079	0.0 (92%)	12	6	1	1
**Pro- and anti-apoptose**							
	**Pro-apoptotic**						
	Caspase 7, apoptosis-related cysteine peptidase	*Xenopus laevis*: NP_001091272	5e-17 (31%)	11	2	2	2
	Cytochrome c	*Pectinaria gouldii*: AAS48105	6e-49 (85%)	1837	880	1	1
	**Anti-apoptotic**						
	Baculoviral IAP repeat-containing protein 3	*Mus musculus*: NP_031490	2e-08 (52%)	20	5	2	2
	Bifunctional apoptosis regulator	*Xenopus tropicalis*: AAI67891	2e-36 (38%)	4	2	1	1
	Baculoviral IAP repeat-containing protein 7	*Xenopus tropicalis*: NP_001106593	2e-08 (47%)	14	5	1	1
	Baculoviral IAP repeat-containing protein 3	*Homo sapiens*: NP_001156	9e-12 (54%)	39	19	1	1
	Inhibitor of apoptosis	*Aplysia californica*: ADI24344	2e-25 (36%)	13	0	1	1
	* Baculoviral IAP repeat-containing protein 4	*Xenopus laevis*: NP_001089083	8e-22 (29%)	24	9	1	1
	Ribonuclease kappa-A	*Danio rerio*: NP_001038870	8e-20 (50%)	20	2	1	1
	Hepatitis B virus x interacting protein	*Gallus gallus*: NP_001165357	9e-14 (44%)	8	1	1	2
**Cellular differentiation**							
	**Signaling molecules**						
	Transforming growth factor-beta-inducible early growth response protein 3	*Salmo salar*: NP_001133301	4e-37 (78%)	29	8	1	1
	Early growth response protein 1	*Ovis aries*: NP_001135978	6e-39 (68%)	5	1	1	1
	Activin type II receptor	*Crassostrea gigas*: CAR92545	0.0 (100%)	37	11	2	2
	Frizzled-5 precursor	*Homo sapiens*: NP_003459	4e-18 (40%)	3	1	1	1
	Frizzled-related protein 2	*Aplysia californica*: AAT07665	1e-62 (51%)	17	4	1	1
	Fizzy-like protein	*Saccoglossus kowalevskii*: NP_001161540	5e-106 (91%)	18	7	1	1
	Calcyclin binding protein	*Xenopus tropicalis*: NP_001011170	3e-28 (43%)	2	0	1	2
	Regucalcin	*Haliotis discus discus*: ABO26616	4e-55 (47%)	2	0	1	1
	**Small GTPase and other regulators**						
	Rho-related GTP-binding protein RhoQ	*Danio rerio*: NP_956112	1e-21 (37%)	5	1	1	1
	Ras-like protein	*Homo sapiens*: AAA36544	1e-94 (91%)	6	3	1	1
	* Rac GTPase-activating protein 1	*Camponotus floridanus*: EFN67432	5e-13 (44%)	20	2	1	1
	Cdc42	*Schistosoma mansoni*: XP_002579369	7e-37 (46%)	23	8	4	5
	Rho family small GTP binding protein cdc42	*Rhopalosiphum padi*: AAS87368	6e-94 (94%)	2	0	1	1
	Small GTPase Cdc42	*Schizophyllum commune*: XP_003036258	7e-63 (60%)	10	4	1	1
	Rab-like protein 3	*Harpegnathos saltator*: EFN90063	6e-64 (57%)	5	2	1	1

	Ras-related protein Rab-6A	*Camponotus floridanus*: EFN73950	1e-93 (93%)	13	5	1	1
	RAB18, member RAS oncogene family	*Xenopus tropicalis*: NP_001017281		22	1	1	1
	GTPase Rab2	*Glossina morsitans morsitans*: ADD20253	4e-46 (50%)	7	0	1	1
	RAN GTPase activating protein 1, isoform CRA_b	*Mus musculus*: EDL04561	2e-07 (51%)	3	0	1	1
	ADP-ribosylation factor GTPase-activating protein, putative	*Pediculus humanus corpori*: XP_002426585	2e-25 (61)	5	1	1	1
	ADP-ribosylation factor-like protein 8A	*Homo sapiens*: NP_620150	3e-94 (91%)	77	31	2	2
	High mobility group protein 20A	*Danio rerio*: NP_001082803	4e-17 (63%)	2	0	1	1
	Coiled-coil domain containing 101	*Xenopus tropicalis*: NP_001120013	7e-107 (68%)	4	2	1	1
	T-box transcription factor TBX20	*Xenopus tropicalis*: NP_001030292	8e-29 (63%)	8	0	1	1
	Tryptophanyl-tRNA synthetase, cytoplasmic	*Bos taurus*: DAA17278	2e-172 (73%)	26	5	1	2
	Serine/threonine protein phosphatase, putative	*Ixodes scapularis*: XP_002414442	6e-13 (100%)	4	2	1	1
	Src tyrosine kinase 2	*Aplysia californica*: ACI23623	6e-103 (88%)	7	0	1	1
	Cyclin G1	*Xenopus laevis*: NP_001090435	9e-22 (34%)	44	22	1	1
	B-cell translocation gene 1 (BTG1)	*Crassostrea gigas*: ACH92125	0.0 (100%)	7	2	1	1
	Origin recognition complex, subunit 1	*Xenopus laevis*: NP_001081806	8e-145 (70%)	12	5	1	1
	Origin recognition complex subunit 3	*Bos taurus*: DAA26171	2e-13 (63%)	2	1	1	1

* Genes used for qPCR analysis.
