# Supplementary figures and images for: Whole Transcriptome Profiling of Successful Immune Response to Vibrio Infections in the Oyster Crassostrea gigas by Digital Gene Expression Analysis
Source: PLoS One. 2011 Aug 4;6(8):e23142. doi: 10.1371/journal.pone.0023142 (PMC3150398; doi:10.1371/journal.pone.0023142)

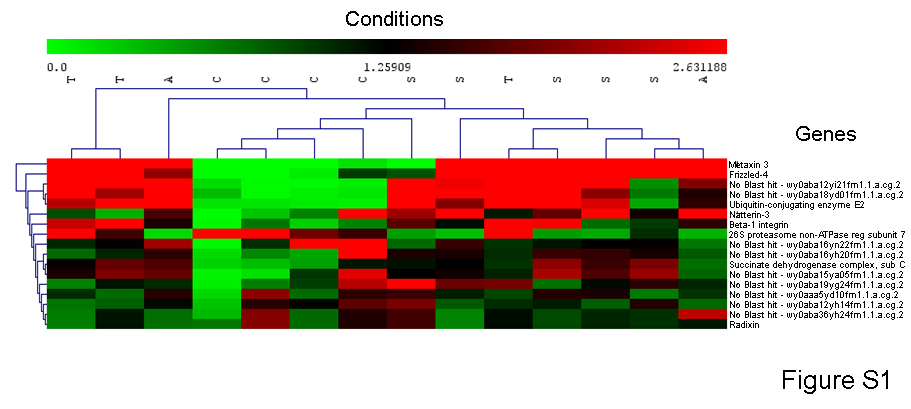

Supplement: Figure S1 — Hierarchical clustering analysis and differential expression of 17 genes from SaVir library. Hemocyte gene expression profiles were analysed in biological replicates from oysters: non injected as control (C), surviving infections with avirulent V. tasmaniensis LMG 20012T (T), with virulent V. splendidus LGP32 (S) and virulent V. aestuarianus LPi 02/41 (A). Each cell in the matrix corresponds to the expression level of one gene in a sample. The intensity of the color from green to red indicates the magnitude of differential expression (see color scale at the bottom of the image). Relative expressions were calculated according the 2−(ΔΔCt) method normalized with elongation factor-1α (EF-1α). Each value was calculated in reference to the mean of ΔCt of all conditions (relative expression = 1). The dendrograms at the top of the figures indicate relationship among experimental conditions which define clusters of conditions (CC). The dendrograms at the left of the figures indicate relationship among the profiles of the selected genes which define clusters of expression (CE), after clustering analysis using Multiple Array Viewer software. (TIF) [file pone.0023142.s004.tif]

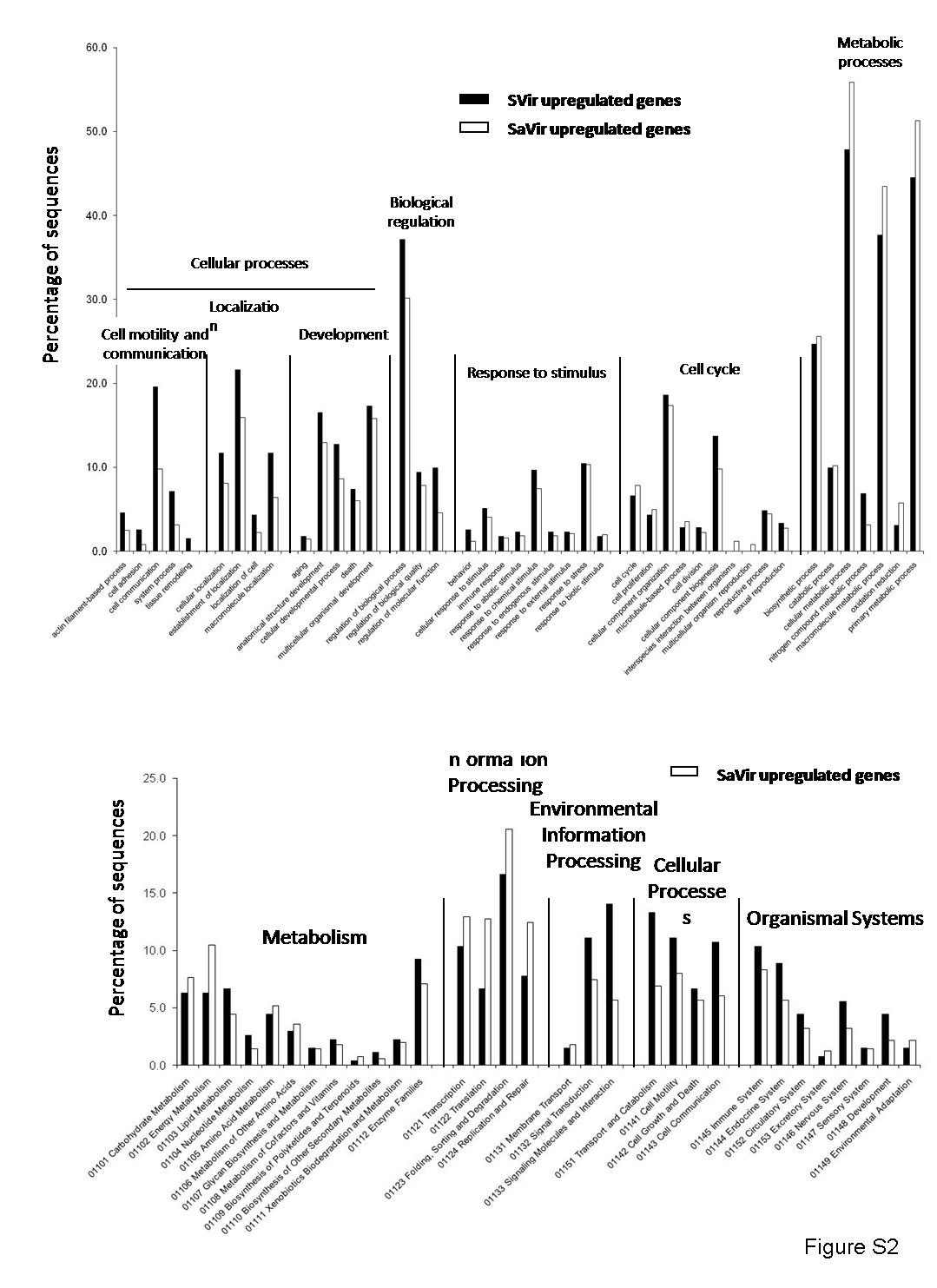

Supplement: Figure S2 — Gene ontology assignment of 1,610 and 2,321 unique ESTs differentially represented between SVir and SaVir libraries, respectively. (a) Categorization based on GO terms assignment from 3nd level of Biological Process using Blast2GO software. (b) Categorization based on KO terms (level A and B) of the Kyoto Encyclopedia of Genes and Genomes (KEGG) using KEGG Automatic Annotation Server (KAAS). (TIF) [file pone.0023142.s005.tif]
